# Preliminary effects of a four-month circuit training intervention on cognitive function and exploratory plasma proteomic profiles in middle-aged and older women: an open-label randomized controlled trial

**DOI:** 10.3389/fspor.2026.1851134

**Published:** 2026-06-23

**Authors:** Keishi Soga, Michio Takahashi, Kentaro Oba, Akari Uno, Takuji Kawamura, Keisei Kawashima, Takamitsu Shinada, Yasuyuki Taki

**Affiliations:** 1 Smart-Aging Research Center, Institute of Development, Aging and Cancer, Tohoku University, Sendai, Japan; 2 Faculty of Humanities, Institute of Human and Social Sciences, Kanazawa University, Kanazawa, Japan; 3 Department of Psychology, Faculty of Humanities, Saitama Gakuen University, Kawaguchi, Japan; 4 Department of Aging Research and Geriatric Medicine, Institute of Development, Aging and Cancer, Tohoku University, Sendai, Japan; 5 Department of Medical Sciences, Graduate School of Medicine, Tohoku University, Sendai, Japan

**Keywords:** aging, cognition, exercise, memory, proteomics

## Abstract

**Background:**

Although exercise can help prevent age-related cognitive decline, the mechanisms underlying its effects on cognition, immune function remain unclear. Therefore, this study comprehensively examined the effects of circuit training on cognitive function, and the plasma proteome in middle-aged to older women.

**Methods:**

Forty-eight women were assigned to either a four-month circuit training program (*n* = 23, exercising more than twice per week) or control group (*n* = 25). Cognitive function was assessed using Stroop, Flanker, N-back, and source memory tasks. Statistical analyses were performed using linear mixed-effects models with Type III analysis of variance. The plasma proteome was measured using Olink technology (1,098 proteins). Proteomic patterns were explored using orthogonal partial least squares discriminant analysis (OPLS-DA). Furthermore, between-group comparisons of protein changes (post-baseline differences) were performed using unpaired *t*-tests. For functional annotation, significantly altered proteins (identified by volcano plot analysis) were subjected to pathway enrichment analyses.

**Results:**

Within-group changes in Stroop interference and d-prime were observed in the circuit training intervention group. Furthermore, the proteins significantly elevated in the exercise intervention group were functionally categorized into anti-inflammatory and immunosuppressive pathways (LRRC25, IL10RA), angiogenesis and tissue remodeling (PROK1, TNFRSF12A), and immune cell activation (CD7). However, no protein–cognition associations remained significant after FDR correction.

**Conclusions:**

This open-label randomized controlled trial provides preliminary evidence that a 4-month circuit training intervention may improve selected cognitive outcomes and alter circulating proteins related to immune, inflammatory, and carbohydrate-binding functions in middle-aged and older women. However, protein–cognition associations did not remain significant after FDR correction. These findings should be interpreted as hypothesis-generating and require confirmation in larger studies with integrated cognitive and molecular analyses.

## Introduction

Physical activity is one of the most promising interventions for preventing age-related cognitive decline and dementia (1–3). Midlife represents a critical window for intervention, as physical activity can influence brain aging while neuroplastic mechanisms remain responsive to exercise-induced adaptations, even in the presence of subtle age-related changes such as synaptic loss and reduced plasticity (4). Meta-analytic evidence demonstrates that long-term exercise interventions improve cognitive function across multiple domains, indicating improved cognitive function following exercise programs, with particularly beneficial effects on executive function and memory (5, 6). Both aerobic exercise and resistance training have been shown to enhance cognitive performance through upregulation of growth factors, including brain-derived neurotrophic factor and insulin-like growth factor, which contribute to neurogenesis and neural plasticity (7, 8). Further, a recent large-scale meta-analysis of 58 cohort and case–control studies demonstrated that habitual physical activity is associated with a 14% reduction in Alzheimer's disease (3). A recent multicenter randomized controlled trial in Japan suggested that higher participation in physical exercise was associated with improved cognitive performance and potentially led to a reduced dementia risk among older adults aged 65–85 years with mild cognitive deficits (9). These findings support physical activity as a modifiable protective factor against cognitive decline across mid-to-late adulthood.

Recent research highlights that physical activity elicits systemic molecular adaptations that may influence brain health through changes in circulating proteins. Comprehensive multiomic profiling from the Molecular Transducers of Physical Activity Consortium (10) revealed exercise-induced alterations in inflammation, mitochondrial metabolism, and stress-response proteins across multiple tissues, including the hippocampus, suggesting coordinated molecular mechanisms linking the peripheral and central systems. In humans, plasma proteomics studies have also demonstrated that exercise modifies proteins related to immune regulation, metabolic control, and muscle development (11). Considering the emerging evidence for the crucial role of immune signaling in maintaining cognitive function and neuronal plasticity (12), exercise-induced changes in circulating immune-related proteins may contribute to neurocognitive benefits. However, the proteomic signatures induced by structured exercise interventions, particularly in the context of cognitive health, remain almost unexplored. Understanding these exercise-induced proteomic changes is essential for identifying novel biomarkers of the exercise response and elucidating the molecular mechanisms underlying the cognitive benefits of physical activity. Characterizing these systemic pathways is key to moving beyond descriptive associations and gaining a comprehensive understanding of how exercise exerts neuroprotective effects.

Therefore, the present study aimed to examine the effects of long-term (i.e., about four-month) circuit training intervention on cognitive function and proteomic profiling. This investigation aimed to comprehensively characterize the biological pathways through which exercise exerts systemic effects. We hypothesized that circuit training enhances cognitive function by modulating immune and inflammatory signaling pathways. Accordingly, this study focused on identifying immune-centered molecular mechanisms by which physical activity promotes systemic homeostasis and supports cognitive resilience.

## Methods

### Study design and participants

**Figure 1:**
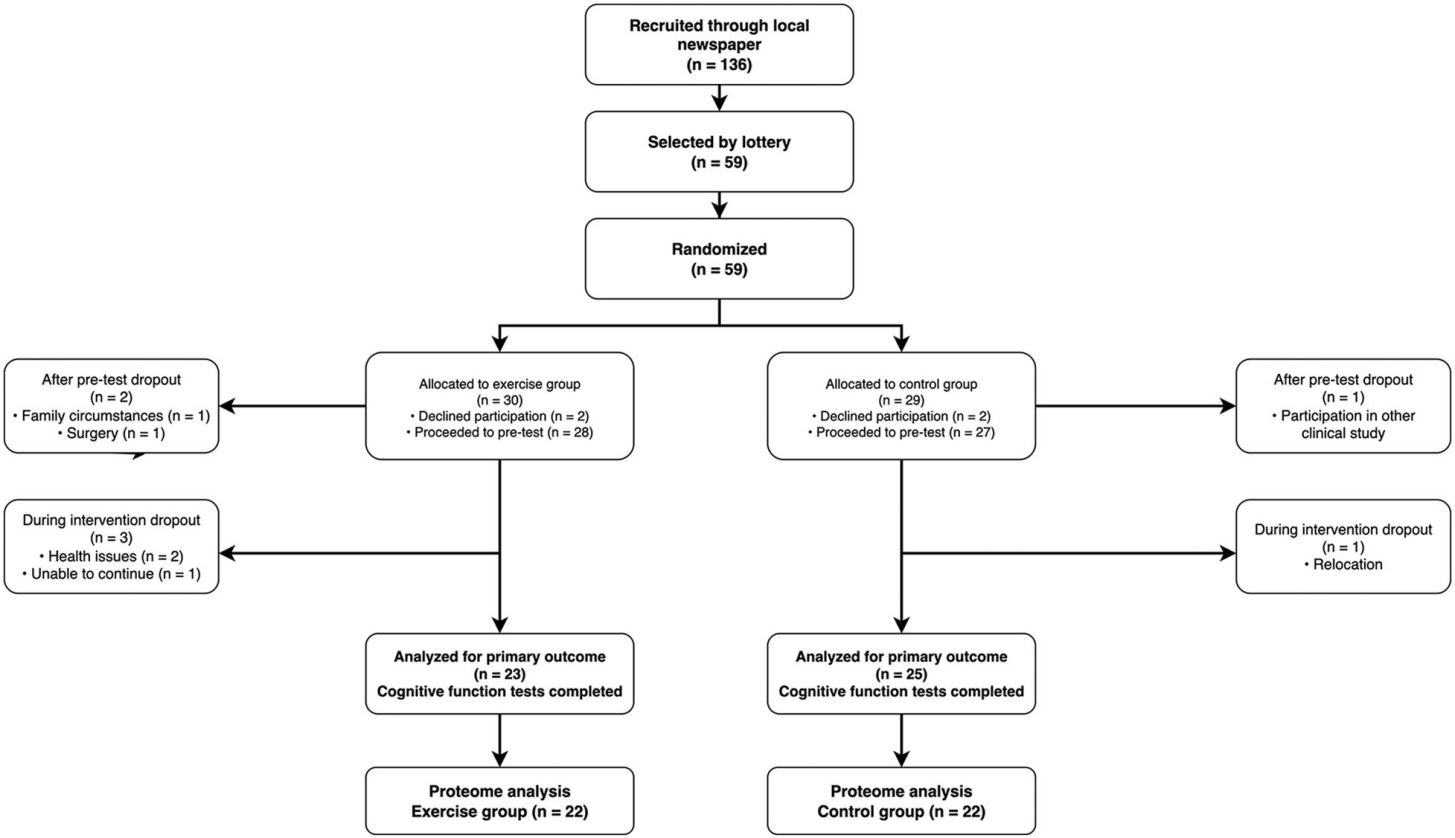
Study flow diagram.

This study, employing a randomized, open-label, controlled trial design, was conducted at Tohoku University, Japan, between June 2024 and February 2025 (UMIN-CTR: UMIN000053937). In total, 59 healthy Japanese women were recruited from the local community and randomly assigned to either a circuit training intervention group (*n* = 30) or waitlist control group (*n* = 29) in a 1:1 ratio using a computer-based random number generator (Research Randomizer, www.randomizer.org). A flowchart of the study is shown in Figure 1. All participants provided written informed consent and the study was approved by the Ethics Committee of Tohoku University Graduate School of Medicine (ID: 2025-1-735). Detailed information on the study design, including the sample size calculation, is provided in the Supplementary Material (Supplementary Methods).

### Procedure

The participants were recruited through advertisements in local community publications distributed near Tohoku University, Japan. The recruitment responses exceeded the target sample size; therefore, prospective participants for the information session were selected from all eligible applicants via a lottery system. The selected individuals were invited to a group information session wherein the study objectives, procedures, and potential risks, and benefits were comprehensively explained. After the session, all eligibility criteria were confirmed and written informed consent was obtained from each participant.

After providing informed consent and completing scheduling coordination, participants underwent baseline assessments on a separate day from the group information session, following a standardized sequence. First, the participants submitted fecal samples self-collected at home using the provided collection kits. Anthropometric measurements (height and weight) were then obtained, followed by the collection of blood samples (approximately 10 mL). Cognitive function was assessed using a comprehensive battery of neuropsychological tests. Physical function tests, including hand grip strength measurement, 5-meter walk test, and single-leg stance tests (under both eyes open and eyes closed conditions), were conducted after cognitive assessment. Upon completion of the baseline assessments, participants were informed of their randomized group assignments. Those assigned to the exercise group were instructed to participate in circuit training sessions at least twice per week for approximately 16 weeks, whereas those in the control group were instructed to maintain their usual daily activities without initiating new, structured exercise programs. Post-intervention assessments were performed following the same protocol and sequence as those used for the baseline measurements after completion of about the 16-week intervention period.

### Statistical analyses

Primary outcomes included cognitive function (executive and memory domains), and proteomic profiles. Secondary outcomes encompassed gut microbiome composition and diversity, physical function, psychological characteristics, mental health indicators, quality of life, physical activity levels, mood states, and sleep quality, as detailed in our study protocol (13). All results related to the secondary outcomes are presented in the Supplementary Material (Supplementary Secondary Outcomes).

Data were preprocessed using Python (version 3.12.0) and all statistical analyses were performed using R (version 4.4.1). Statistical significance was set at *α* = 0.05. Data are presented as the mean ± standard deviation unless otherwise specified.

Cognitive Performance: Linear mixed-effects models were used to analyze changes in cognitive performance from the baseline to post-intervention (16 weeks). The models included fixed effects for group (exercise vs. control), time (baseline vs. post), and group × time interactions, with participant as a random effect. For exploratory statistical analysis, a Type III analysis of variance (ANOVA) was performed on the linear mixed-effects models to assess the significance of fixed effects and guide further investigation. When a significant main effect of time or group × time interaction was observed, *post-hoc* comparisons were conducted using estimated marginal means (EMMs) with Bonferroni adjustment to compare the pre- and post-intervention scores within each group. The EMMs approach provides model-based estimates of group means, while accounting for the random-effects structure and unbalanced data. The EMMs and their standard errors were obtained using the emmeans package.

Protein Biomarkers: For multivariate analysis, orthogonal partial least-squares discriminant analysis (OPLS-DA) was used to maximize the separation between groups (Intervention vs. Control groups) and to identify potential variables contributing to this separation, following a previous proteomic study that investigated the effects of exercise (11). To identify proteins differentially regulated between the Intervention and Control Groups, we performed OPLS-DA using the pre-to-post changes (ΔNPX values). Data were scaled using standard scaling (mean centering and unit variance scaling). OPLS-DA was performed with one predictive component and orthogonal components determined automatically, using seven-fold cross-validation. Feature selection was based on two criteria (1): Variable Importance in Projection (VIP) score ≥ 1.0, indicating a substantial contribution to group separation, and (2) absolute correlation with the predictive component [|p(corr)| ≥ 0.35], reflecting the reliability of the association between the features and group classification. For proteins selected using OPLS-DA, we compared the magnitude of pre-to post-treatment changes between groups using Welch's *t*-test, which does not assume equal variance. The mean ΔNPX values were calculated for each group, and the difference between groups was expressed as both log2 difference (mean Δ Intervention Group – mean Δ Control Group) and fold change (2^log2^ difference). *P*-values were adjusted for multiple testing using the Benjamini–Hochberg false discovery rate (FDR) method. Proteins with FDR-adjusted q-values < 0.05 were considered statistically significant. For sensitivity analysis, Welch's *t*-tests were conducted across all measured proteins to compare changes in NPX values between the exercise and control groups. Benjamini–Hochberg FDR correction was applied across all tested proteins.

## Results

### Participant characteristics and study completion

In total, 51 participants were recruited and randomized to either the exercise intervention group (*n* = 25) or control group (*n* = 26). The baseline characteristics of the participants are summarized in Table 1. Of the 51 enrolled participants, 48 (94%) completed the 16-week study (exercise group, *n* = 23; control group, *n* = 25). Participants in the intervention group were encouraged to attend at least two circuit training sessions per week. Because some participants attended more than two sessions per week depending on their availability, adherence was evaluated based on the total number of completed sessions over the intervention period rather than a fixed weekly frequency. Although the intervention was designed as a 16-week program, the actual intervention period ranged from 16 to 18 weeks because of scheduling adjustments for post-intervention assessments. Therefore, the maximum planned number of sessions was defined as 36 sessions, corresponding to two sessions per week for 16 to18 weeks. Adherence was defined as attendance at ≥80% of the planned sessions, equivalent to at least 29 sessions. All 23 completers in the intervention group attended at least 29 sessions and therefore met the predefined adherence criterion of ≥80% attendance. The actual number of completed sessions was 44.55 ± 11.3 sessions.

**Table 1:** The baseline characteristics of the participants.

Variable	Control group Mean (SD)	Intervention group Mean (SD)	*p*-value
Age (years)	59.12 (9.49)	58.78 (8.30)	0.90
Weight (kg)	54.21 (8.62)	55.73 (11.69)	0.61
Height (cm)	157.12 (4.53)	156.24 (5.29)	0.54
Education (years)	14.04 (1.94)	14.43 (1.80)	0.48

### Cognitive tasks

#### The Stroop task

**Figure 2:**
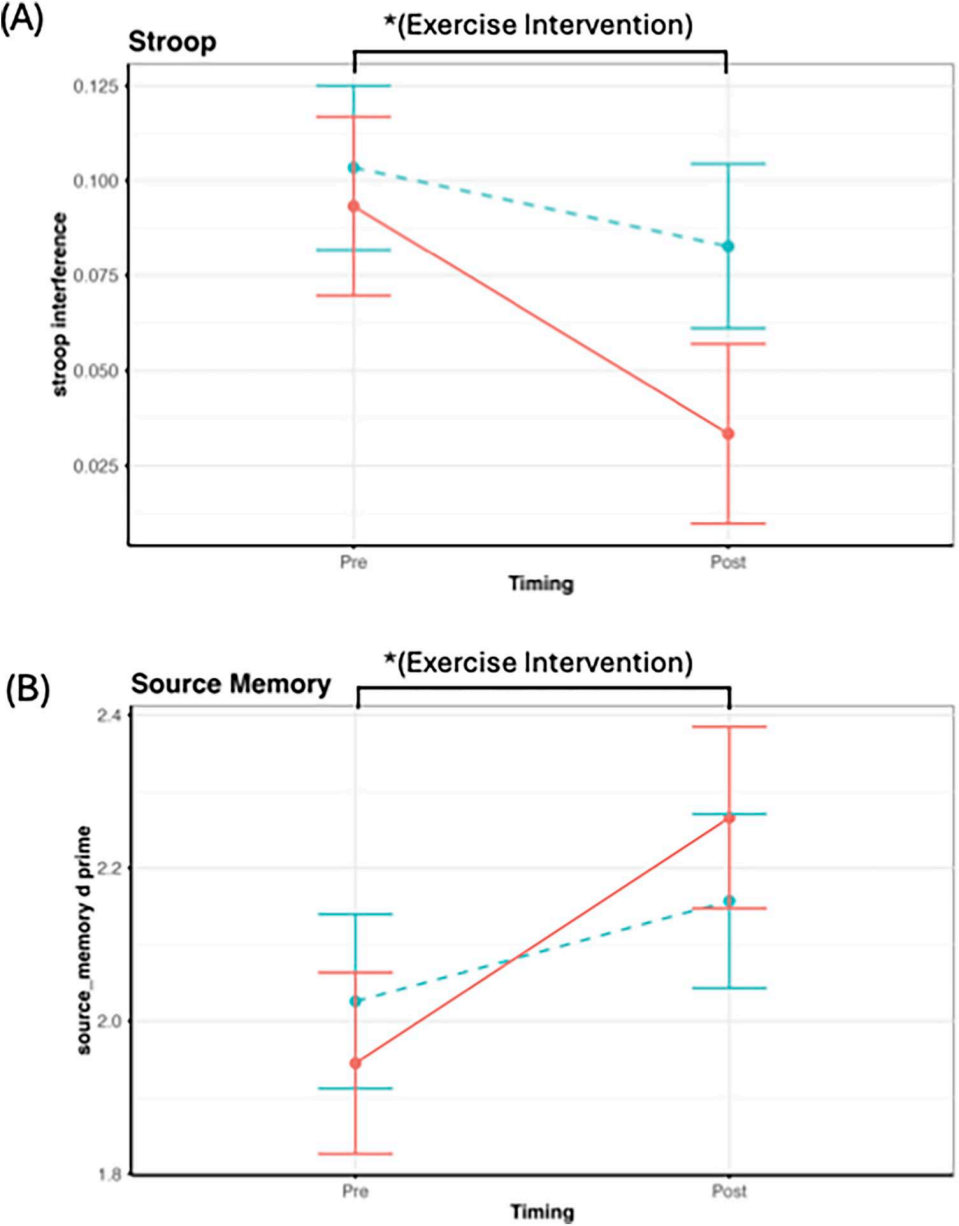
Changes in cognitive performance in the Stroop task **(A)** and the source memory task **(B)**. The green dashed line represents the control group, and the red solid line represents the exercise intervention group. *: *p* < 0.05.

Linear mixed-effects model analysis revealed no significant group × time interactions for either the Stroop interference scores (*p* = 0.283) or the reverse Stroop interference scores (*p* = 0.653). However, a significant main effect of time was observed for Stroop interference [F_(1, 46)_ = 4.97, *p* = 0.031] in the ANOVA model, indicating an overall improvement in inhibitory control across the study period. *Post-hoc* within-group comparisons showed that the exercise group demonstrated a significant reduction in Stroop interference from the baseline (0.095 ± 0.022) to post-intervention (0.039 ± 0.022, *p* = 0.033, d = 0.66), suggesting a preliminary within-group change in inhibitory control. In contrast, the control group showed no significant change in interference scores (baseline: 0.103 ± 0.021; post-intervention: 0.083 ± 0.021, *p* = 0.406, d = 0.24). The results are presented in Figure 2A.

#### Flanker task

The linear mixed-effects model analysis revealed no significant group × time interactions for any of the measured outcomes (all *p* > 0.05). Although significant main effects of time were observed in the ANOVA models for both congruent [F _(1,46)_ = 5.44, *p* = 0.024] and incongruent reaction times [F _(1,46)_ = 5.37, *p* = 0.025], *post-hoc* within-group comparisons showed no significant changes from the baseline to post-intervention in either group for congruent reaction time or incongruent reaction time.

#### N-back task

For both the 1-back and 2-back conditions, the linear mixed-effects model analysis revealed no significant group × time interactions for any of the measured outcomes (all *p* > 0.05). In the 2-back condition, a significant main effect of time was observed in the ANOVA models for the false alarm rate [F (1,45) = 4.75, *p* = 0.035] and correct rejection rate [F (1,45) = 4.75, *p* = 0.035]. Based on our hypothesis, *post-hoc* within-group comparisons showed no significant changes from the baseline to post-intervention in either group.

### Episodic memory

#### Source memory task

A linear mixed-effects model analysis revealed no significant group × time interactions for any of the measured outcomes (all *p* > 0.05). However, a significant main effect of time was observed for the hit rate (t = 3.064, *p* = 0.004) and miss rate (t = 3.064, *p* = 0.004) in the linear mixed-effects model. Further, significant main effects of time were observed in the ANOVA models for hit rate [F _(1, 48)_ = 20.40, *p* < 0.001], and d-prime [F _(1, 48)_ = 12.11, *p* = 0.001], indicating overall improvements in episodic memory performance across the study period.

Based on our hypothesis, *post-hoc* within-group comparisons were performed to descriptively examine changes from baseline to post-intervention. Hit rate showed significant within-group increases in both groups (exercise group: 68.8 ± 2.9% to 76.9 ± 2.9%, *p* = 0.002, d = 0.98; control group: 73.6 ± 2.8% to 80.7 ± 2.8%, *p* = 0.004, d = 0.87). For source memory correct scores, a significant within-group increase was observed in the exercise group (baseline: 22.0 ± 1.8; post-intervention: 24.9 ± 1.8, *p* = 0.047, d = 0.62), whereas the control group showed a non-significant trend toward an increase (baseline: 22.7 ± 1.7; post-intervention: 25.3 ± 1.7, *p* = 0.059, d = 0.56). For d-prime scores, the exercise group showed a significant within-group increase (baseline: 1.94 ± 0.12; post-intervention: 2.27 ± 0.12, *p* = 0.002, d = 1.01), whereas the control group showed no significant change (baseline: 2.03 ± 0.11; post-intervention: 2.16 ± 0.11, *p* = 0.160, d = 0.41). The results are presented in Figure 2B.

### Proteomic analysis

**Figure 3:**
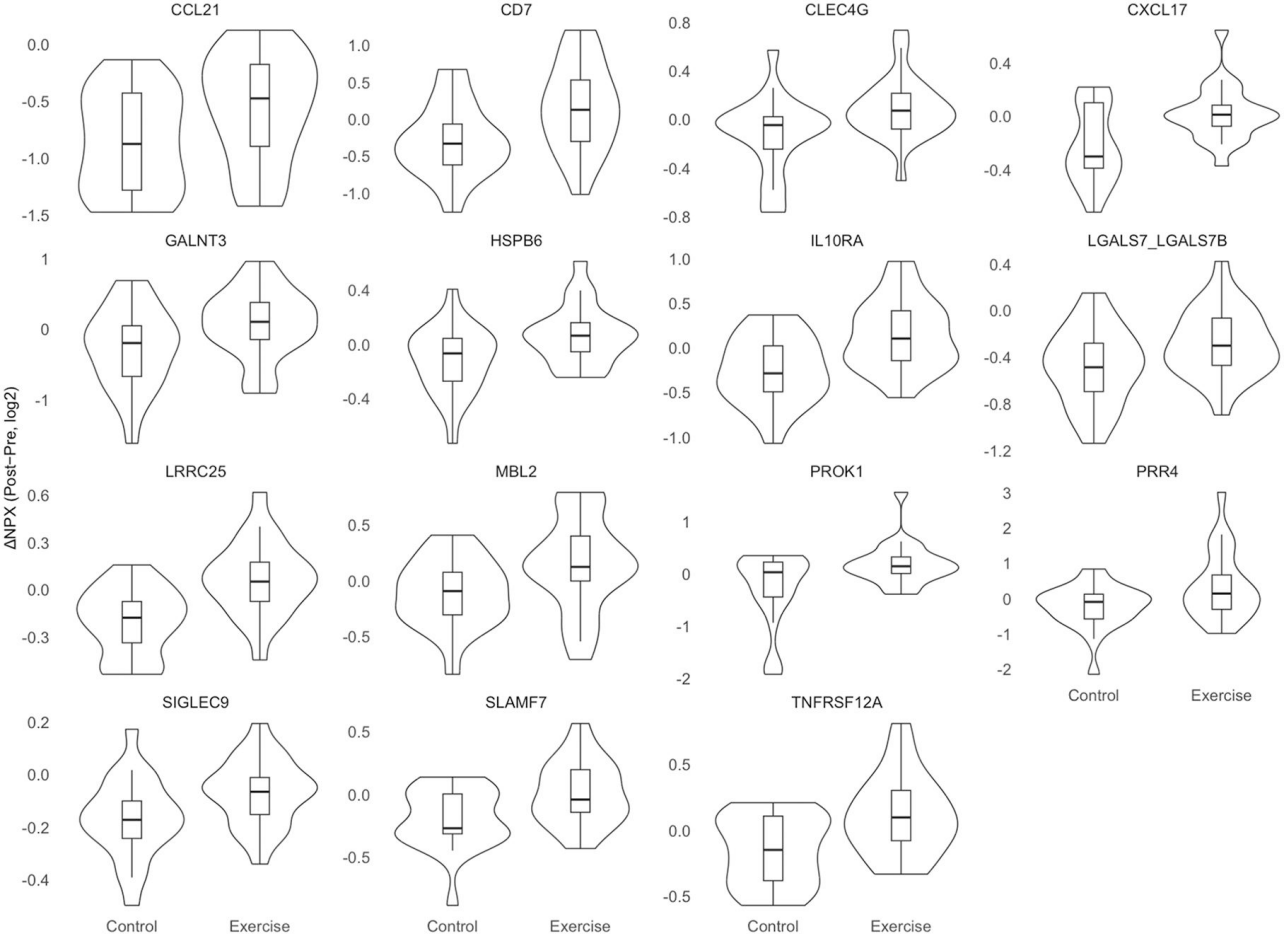
Comparison of changes in protein expression between the exercise and control groups based on orthogonal partial least squares discriminant analysis–selected features [Variable importance in projection ≥ 1 and |p(corr)| ≥ 0.35]. Violin plots show ΔNPX values (Post – Pre, log_2_ scale) for the top 15 proteins that significantly changed (false discovery rate < 0.05). Each panel represents one protein assay, with the control group shown on the left and the exercise group on the right. Protein names are arranged in alphabetical order.

To explore the plasma protein changes differentiating the intervention group from the control group, an OPLS-DA was performed on the pre-to-post changes (ΔNPX values) for all participants. The resulting OPLS-DA model yielded one predictive and two orthogonal components. The model quality parameters were R^2^ = 0.946 and Q^2^ = 0.252. Although the model showed high explanatory power for the current dataset (R^2^), its low predictive ability (Q^2^) suggested that the separation between the two groups was weak and that the model should be considered exploratory. Based on the feature selection criteria [VIP **≥** 1.0 and absolute correlation |p(corr)| **≥** 0.35], a total of 58 proteins were identified as potentially contributing to the group separation observed in this model. These 58 proteins were further analyzed using Welch's *t*-test with FDR correction to identify statistically significant differences. This analysis identified 15 proteins that were significantly upregulated in the exercise intervention group. Among the 15 proteins upregulated in the exercise intervention group, the top hits were LRRC25 [Fold Change (FC) = 1.21, q = 0.014], IL10RA (FC = 1.32, q = 0.023), PROK1 (FC = 1.40, q = 0.025) and CD7 [Fold Change (FC) = 1.37, q = 0.026] (Figure 3). Permutation testing with 1,000 permutations was performed to evaluate the robustness of the OPLS-DA model. The model showed R^2^Y(cum) = 0.946 and Q^2^(cum) = 0.252. The permutation test yielded pR^2^Y = 0.152 and pQ^2^ = 0.005. Although the Q^2^ permutation test suggested that the model performed better than expected by chance, the relatively low Q^2^ value indicated limited cross-validated predictive performance. As a sensitivity analysis, Welch's *t*-tests were performed across all measured proteins, followed by Benjamini–Hochberg FDR correction across all tests. No proteins remained statistically significant after correction across all measured proteins (FDR < 0.05). Therefore, the OPLS-DA-derived proteins were interpreted as exploratory candidate proteins for downstream hypothesis-generating analyses. Detailed results of serum proteomic profiling for the exercise and control groups are provided in the Supplementary Results (Supplementary Proteome Results).

Exploratory protein-cognition association analyses were performed to examine whether changes in the 15 OPLS-DA-derived candidate proteins were associated with changes in cognitive outcomes. No associations between changes in Stroop interference and changes in candidate proteins remained significant after FDR correction in the full sample. In the exercise-group-only analysis, GALNT3 (*p* = 0.0247, q = 0.371) showed a nominal association with changes in Stroop interference, but this association did not survive FDR correction. For source memory outcomes, HSPB6 (*p* = 0.00427, q = 0.641) and SIGLEC9 (*p* = 0.00214, q = 0.160) showed the strongest nominal association with changes in d-prime within the exercise group, but this association also did not remain significant after FDR correction. These results did not provide robust evidence of direct protein-cognition associations in the present sample. To further characterize the biological relevance of the OPLS-DA-derived candidate proteins, we performed exploratory GO and KEGG enrichment analyses. GO enrichment analysis identified one significantly enriched Molecular Function term, carbohydrate binding (GO:0030246; GeneRatio = 5/15; Fold Enrichment = 6.74; adjusted *p* = 0.0226), involving GALNT3, CLEC4G, SIGLEC9, LGALS7B, and MBL2. No KEGG pathways remained statistically significant after FDR correction.

## Discussion

The present study investigated preliminary changes in selected cognitive outcomes and exploratory plasma proteomic profiles following a four-month circuit training intervention in middle-aged and older women. We sought to elucidate the complex mechanisms underlying exercise-induced physiological adaptations and cognitive improvements. Following an approximately four-month circuit training intervention, preliminary within-group changes were observed in selected cognitive outcomes, including Stroop interference and d-prime, whereas working memory remained unchanged. In addition, proteomic analysis of OPLS-DA identified specific protein biomarkers, such as anti-inflammatory and immunosuppressive pathways (e.g., LRRC25 and IL10RA), angiogenesis and tissue remodeling (e.g., PROK1 and TNFRSF12A), and immune cell activation (e.g., CD7). However, no proteins remained significant when FDR correction was applied across all measured proteins. This finding underscores the exploratory nature of the proteomic results and indicates that the OPLS-DA-derived proteins should not be interpreted as definitive differentially expressed proteins. The additional enrichment analysis suggested that the OPLS-DA-derived candidate proteins were enriched for carbohydrate binding. However, KEGG pathway analysis did not identify significant pathway-level enrichment, and the exploratory protein-cognition correlation analyses did not reveal robust associations after FDR correction. Therefore, the present proteomic findings should be interpreted as exploratory and hypothesis-generating rather than as definitive evidence of integrated biological pathways underlying cognitive improvement. By comprehensively examining the cognitive and proteomic systems, this study provides a perspective on the biological mechanisms of exercise adaptation. Elucidating these mechanisms could facilitate the development of targeted, mechanism-based exercise interventions that account for individual variability in immune, metabolic, and neural responses.

The cognitive findings should be interpreted as exploratory and potentially domain-specific, as preliminary within-group changes were observed in inhibitory control and episodic memory, whereas working memory remained unchanged. Improvement in Stroop task performance, reflecting enhanced inhibitory control, aligns with previous meta-analyses demonstrating that long-term exercise interventions benefit executive functions (5, 14, 15). Notably, although the Stroop task showed significant improvement, the computerized Flanker task did not, suggesting that different measures of inhibitory control may be differentially sensitive to exercise-induced changes. This finding highlights the importance of using multiple assessment tools when evaluating cognitive outcomes. Preliminary within-group changes in episodic memory, reflected by changes in source memory accuracy and d-prime scores, may extend previous findings by suggesting that circuit training could be associated with changes in both executive and memory-related cognitive domains. The absence of improvements in working memory (N-back tasks) may reflect the specific demands of the exercise intervention or the sensitivity of different cognitive domains to various types of physical activity. Consistent with previous meta-analytical evidence, exercise interventions appear to have limited effects on working memory. Both aerobic exercise and resistance training have been reported to produce no significant improvements in working memory performance (16, 17). Taken together, these findings suggest that exercise preferentially strengthens the neural systems supporting inhibitory control and episodic memory, whereas working memory may be less sensitive to training effects. This reduced sensitivity can be attributed to task-specific demands or ceiling effects associated with relatively simple paradigms.

Furthermore, the absence of improvement in N-back performance may indicate that the cognitive effects of circuit training were domain-specific. The task-specific pattern of cognitive changes may be partly explained by differences in the cognitive processes and neural systems engaged by each task. The N-back task places high demands on working memory processes, including online monitoring, updating, and manipulation of information, and a quantitative meta-analysis of functional neuroimaging studies showed that N-back paradigms consistently activate a distributed frontoparietal working-memory network, including the dorsolateral and ventrolateral prefrontal cortices and posterior parietal cortices (18). In contrast, source memory requires the retrieval of contextual information associated with an item, and fMRI studies have implicated frontal regions and medial temporal structures, including the hippocampal formation and medial frontal cortex, in successful source-memory retrieval (19). Stroop interference, meanwhile, reflects inhibitory control and conflict monitoring. A fMRI study using a Stroop paradigm demonstrated dissociable roles of the left dorsolateral prefrontal cortex and anterior cingulate cortex, with the former supporting the implementation of cognitive control and the latter contributing to performance or conflict monitoring (20). Therefore, the absence of improvement in N-back performance despite improvements in source memory and Stroop interference may reflect the fact that these tasks rely on partly distinct neural systems and cognitive processes.

Regarding proteomic profiles, a key finding from our multivariate analysis (OPLS-DA) was the identification of LRRC25 (Leucine-rich repeat-containing protein 25), IL10RA (Interleukin-10 receptor subunit alpha), PROK1 (Prokineticin-1), CD7 (T-cell antigen CD7), and TNFRSF12A (Tumor necrosis factor receptor superfamily member 12A) as top-ranking proteins significantly elevated in the intervention group compared with those in the control group. These proteins are involved in anti-inflammatory and immunosuppressive pathways (e.g., LRRC25 and IL10RA), angiogenesis and tissue remodeling (e.g., PROK1 and TNFRSF12A), and immune cell activation (e.g., CD7). Notably, our findings correspond with previous suggestions (21), indicating that habitual exercise induces anti-inflammatory responses. This exercise-induced modulation of the immune system is thought to be a key mechanism contributing to overall health maintenance. As demonstrated in a large-scale systematic review and meta-analysis (22), regular physical activity is associated with a reduced risk of community-acquired infectious diseases. Improvements in immune function have been proposed to support brain health, and lifestyle factors, such as physical exercise, have been identified as potential modulators of this process (12).

The present findings suggest that circuit training may influence peripheral immune- and inflammation-related proteins. However, whether these changes are directly linked to central nervous system adaptations remains unclear. Although the observed changes in immune- and inflammation-related proteins may suggest systemic biological adaptations to circuit training, the present study cannot determine whether these peripheral proteomic changes directly contributed to central nervous system processes. We did not assess neuroimaging outcomes, cerebrospinal fluid biomarkers, blood-brain barrier permeability, or other direct markers of brain inflammation or neuroplasticity. Therefore, the observed protein changes may reflect adaptations in skeletal muscle, circulating immune cells, vascular tissues, or other peripheral systems rather than direct effects on the brain. Future studies incorporating neuroimaging, cerebrospinal fluid biomarkers, blood-brain barrier-related assessments, or experimental animal models are needed to clarify whether exercise-induced peripheral proteomic changes are mechanistically linked to central nervous system adaptations and cognitive outcomes.

Previous large-scale plasma proteomic studies have shown that endurance exercise training induces broad changes in circulating proteins (23) reporting that a 20-week endurance exercise training intervention altered hundreds of plasma proteins, including proteins related to angiogenesis, iron homeostasis, extracellular matrix remodeling, and secreted factors associated with cardiorespiratory fitness adaptations. These findings support the concept that regular exercise can modulate circulating proteomic profiles. However, compared with endurance exercise, the circulating proteomic responses to resistance training remain less well characterized. In particular, it remains unclear which circulating proteins are specifically altered by resistance exercise or by combined exercise modalities that include both aerobic and resistance components. Therefore, the present proteomic analysis was conducted as an exploratory investigation to generate hypotheses regarding potential circulating protein changes associated with circuit training.

This study has some limitations that warrant consideration when interpreting its findings. First, the relatively small sample size (*n* = 44 for proteomic analysis) may curtail generalizability, especially for exploratory analyses that probe inter-individual variability in microbiota–cognition associations. Our OPLS-DA model used for feature selection demonstrated a low predictive ability (Q^2^ = 0.252) despite its high explanatory power (R^2^ = 0.946). This discrepancy suggests the possibility of overfitting. This is attributable to the high-dimensional nature of our data with a large number of protein variables relative to a small sample size. Although permutation testing suggested that the OPLS-DA model's Q^2^ value was unlikely to have arisen by chance, the absolute Q^2^ value was relatively low. This indicates limited cross-validated predictive performance and suggests that the model may not generalize well beyond the present dataset. Given the modest sample size and high-dimensional proteomic data, the OPLS-DA-derived proteins should be interpreted as exploratory candidate proteins rather than validated biomarkers or robust predictive signatures. The low predictive performance may also suggest that biological responses to circuit training differ across individuals. If confirmed in larger studies, such heterogeneity would indicate that a uniform exercise prescription may not be optimal for all participants. Future studies with larger samples and independent validation cohorts are needed to identify responder and non-responder profiles and to examine whether baseline characteristics, adherence, physiological responses, or molecular signatures can predict cognitive and proteomic responses to exercise. Furthermore, although circuit training was associated with improvements in selected cognitive outcomes and changes in circulating proteins, the additional exploratory correlation analyses did not identify robust protein-cognition associations after correction for multiple testing. Therefore, the present study does not establish that peripheral proteomic changes mediated or directly explained the cognitive improvements. The cognitive and proteomic findings should instead be interpreted as exploratory observations that occurred in parallel. In addition, d-prime was calculated as an exploratory measure of item recognition sensitivity. Boundary corrections were applied to avoid undefined z-values, as described in the Supplementary Methods. Although this correction was applied consistently across all participants, the resulting d-prime values should be interpreted as approximate exploratory indices. Future studies with larger samples, prespecified cognitive and molecular endpoints, appropriate handling of multiple testing, and validated measures of memory sensitivity are needed to clarify whether exercise-induced proteomic changes are mechanistically linked to cognitive adaptations. This study also focused exclusively on a female cohort to account for sex-specific hormonal and immune profiles in proteomics. Although this design enabled a more homogeneous investigation of biological pathways, it limits the generalizability of our findings, and future research including male participants is necessary to provide a broader understanding of the exercise–brain–gut axis. In addition, gut microbiome, psychological characteristics, and sleep quality were collected as exploratory secondary outcomes but were not incorporated into the main cognitive-proteomic analyses. Thus, the present study cannot determine whether these factors contributed to the observed cognitive or proteomic changes. Future studies should integrate these multidomain outcomes using prespecified analytical frameworks. Finally, the absence of dietary control and potential seasonal fluctuations in the gut microbiota composition constitute additional confounding factors that may have influenced the outcomes.

In conclusion, this open-label randomized controlled trial provides preliminary evidence that a Four-month circuit training intervention may improve selected cognitive outcomes and alter circulating proteins related to immune, inflammatory, and carbohydrate-binding functions in middle-aged and older women. However, the proteomic findings were exploratory, and the present study did not establish a causal or mediating role of these proteins in cognitive improvement. Larger studies integrating cognitive outcomes, proteomic profiling, pathway-level analyses, and central nervous system biomarkers are needed to clarify the biological mechanisms underlying exercise-related cognitive adaptations.

## Data Availability

De-identified data and analytic code are available from the corresponding author upon justified request.
