# Supplementary material for: Preliminary effects of a four-month circuit training intervention on cognitive function and exploratory plasma proteomic profiles in middle-aged and older women: an open-label randomized controlled trial
Source: Front Sports Act Living. 2026 Jun 23;8:1851134. doi: 10.3389/fspor.2026.1851134 (PMC13337939; doi:10.3389/fspor.2026.1851134)
Supplement: Supplementary file 1 [file Supplementaryfile1.docx]

**Inclusion and exclusion criteria**

The inclusion criteria were as follows: (1) native Japanese speakers aged 40–74 years; (2) female sex; (3) ability to participate in exercise sessions 2–3 times per week for 16 weeks; and (4) provision of informed consent. The exclusion criteria were as follows: (1) presence of nervous system diseases; (2) use of medications affecting cognitive function (Antiarrhythmic medication was not an exclusion criterion, provided that the participant's physician had cleared them for exercise); (3) serious medical conditions contraindicating exercise; (4) severe visual or auditory impairments; (5) regular engagement in structured exercise; (6) pregnancy or nursing; and (7) participation in other clinical studies. All participants provided written informed consent and the study was approved by the Ethics Committee of Tohoku University Graduate School of Medicine (ID: 2023-1-1016).

**Intervention**

Participants in the intervention group completed an approximately 16-week circuit training program developed by Curves Japan Co., Ltd. and attended sessions more than two times per week. Each 30-minute session consisted of 12 resistance exercises alternating with aerobic activity, followed by stretching. Resistance exercises included chest press/seated row, squats, shoulder press/lat pull, leg extension/leg curl, abdominal crunch/back extension, lateral lift, elbow flexion/extension, horizontal leg press, pectoral deck, oblique, hip abductor/adductor, and gluteus exercises using pneumatic resistance machines. Aerobic intervals involved floor-based activities, such as stepping or marching in place. Training intensity was maintained at moderate to vigorous levels (60–80% maximum heart rate). The control group maintained their usual daily activities without initiating any new structured exercise programs.

**Selective Attention and Response Inhibition**

The Stroop task was administered using a paper-and-pencil version to assess inhibitory control [1]. The task included four conditions: (1) Neutral Condition 1, where participants identified color words (e.g., "RED") printed in black ink; (2) Reverse-Stroop Condition, where participants identified the word meaning (e.g., "RED") when presented with incongruent color-word combinations (e.g., the word "RED" printed in blue ink); (3) Neutral Condition 2, where participants identified colored patches; and (4) Stroop Condition, where participants identified the ink color rather than the word meaning in incongruent color-word combinations. Participants were instructed to respond as quickly and accurately as possible within 60 seconds, with the primary outcome being the number of correct responses. For the Stroop task analysis, two participants were excluded from the assessment: one due to a procedural error involving an incorrect measurement duration, and one due to an inability to follow the task instructions. Consequently, the final analysis included 21 participants in the exercise group and 25 in the control group (n = 46).

The computerized Flanker task was used to assess inhibitory control using PsychoPy software. Participants viewed arrays of five arrows and were required to indicate the direction of the central target arrow by pressing "c" (left) or "m" (right) keys. The task included 100 trials under congruent (> > > > >, < < < < <) and incongruent (< < > < < , > > < > > ) conditions. Each array was displayed for 300 ms, followed by a fixed inter-stimulus interval of 1400 ms. Primary outcomes included accuracy (percentage of correct responses) and reaction time (milliseconds). For the computerized flanker task analysis, two participants were excluded from the assessment: one because of a button operation error resulting in no responses and one because of an inability to perform the task (failure to remember the task instructions). The final analysis included 23 participants in the exercise group and 23 in the control group (n = 46).

**Working Memory**

Working memory capacity was evaluated using computerized N-back tasks (1-back and 2-back conditions) implemented in PsychoPy. Participants viewed single digits (1–9) and were required to indicate whether the current stimulus matched the stimulus presented n trials back. In the 1-back condition, participants pressed "m" if the current number matched the previous number, otherwise "c.” In the 2-back condition, participants pressed "m" if the current number matched the number presented two trials earlier, otherwise "c.” Each condition comprised 90 trials with a 30% target probability. Stimuli were presented for 2000 ms following a 500 ms fixation period. Primary outcomes included accuracy (percentage correct) and reaction time (ms). For the 1-back task analysis, one participant was excluded because of extremely poor performance at the baseline, suggesting failure to understand the task instructions. The final analysis included 23 and 24 participants in the exercise and control groups, respectively (n = 47). For the 2-back condition, three participants were excluded: two because of an inability to perform the task at the baseline and one because of an inability to perform the task at post-intervention. The final analysis included 22 and 23 participants in the exercise and control groups, respectively (n = 45).

**Episodic Memory**

Memory function was assessed using a computerized source memory task comprising the encoding and retrieval phases, according to a previous study paradigm [2]. During encoding, participants viewed 72 black-and-white outline pictures presented randomly in one of the four quadrants of a grid layout. Participants were instructed to memorize the pictures and judge whether each depicted a natural or an artificial object. Pictures were displayed for 1500 ms with 2500 ms inter-stimulus intervals. During retrieval, participants viewed 108 pictures (72 old, 36 new) and made old/new recognition judgments by pressing "c" (new) or "m" (old). For items judged as "old," the participants indicated the quadrant spatial location using keys "v," "f," "n," or "j." Primary outcomes included recognition accuracy (hit rate), false alarm rate, discriminability (d-prime), and source memory accuracy (correct location identification for correctly recognized old items). The d-prime value was calculated as the difference between the z-transformed hit rate and false alarm rate for old/new item recognition. Hit and false alarm rates were first converted from percentages to proportions. Consistent with previous studies [3], to avoid undefined values at the boundaries, proportions of 0 and 1 were replaced with 0.01 and 0.99, respectively. This correction was applied consistently across all participants. Therefore, this analysis was interpreted as an exploratory measure of item recognition sensitivity.

**Gut Microbiota Analysis**

Fecal samples were collected at the baseline and post-intervention using Metabolokeeper® preservation solution (TechnoSuruga Laboratory, Shizuoka, Japan). DNA was extracted, followed by amplicon sequencing, with PCR amplification targeting the V3-V4 region of the 16S rRNA gene using the Illumina MiSeq system. Data processing was performed using the QIIME2 software with taxonomic assignment based on the Greengenes database. Measures included alpha diversity (Shannon index, observed species), beta diversity (weighted and unweighted UniFrac distances), and the relative abundance of bacterial taxa. Short-chain fatty acid (SCFA) concentrations were determined via physicochemical analysis of fecal samples.

**Proteomic Analysis**

Blood samples (approximately 10 mL) were collected at the baseline and postintervention for comprehensive proteomic analysis. After collection, the samples were centrifuged at 2200 × *g* for 15 min to separate the plasma. The separated plasma was aliquoted into microtubes as follows: at the baseline, five aliquots (150 μL × 1, 250 μL × 2, 500 μL × 2); at post-intervention, four aliquots (250 μL × 2, 500 μL × 2). One 250 μL aliquot from each time point was used for proteomic analysis. All samples were stored at -80°C until analyzed. Proteomic analysis was conducted by RIKEN GENESIS using the Olink® Explore platform. Three protein panels were used: Olink® Explore 384 Inflammation, Olink® Explore 384 Inflammation II, and Olink® Explore 384 Oncology, allowing quantitative measurement of up to 1152 unique proteins. Olink technology utilizes a Proximity Extension Assay (PEA), which employs paired antibodies to ensure high specificity and sensitivity for protein detection across diverse biological pathways, including immune function, inflammation, metabolism, cell signaling, and stress responses. Raw data from the assay (counts) were subjected to quality control (QC) and a multistep normalization process according to the manufacturer's protocol to generate Normalized Protein expression (NPX) values. This process involved normalization against an internal Extension Control, followed by log2 transformation and subsequent between-plate normalization to minimize technical variation. The resulting NPX values represented the relative quantification of protein levels on a log2 scale and were used for all subsequent statistical analyses. Proteomic quality control was performed according to Olink’s standard quality control procedures. According to the project-specific Olink QC report, all 88 samples passed sample QC across the Explore 384 Inflammation, Inflammation II, and Oncology panels. The proportions of datapoints passing QC were 100%, 99%, and 100%, respectively. Thirty assays did not meet Olink’s quality control criteria for batch release and were not included in this project. In addition, NPX Explore HT & 3072, does not calculate limit of detection (LOD) values. Therefore, no proteins were excluded on the basis of being below the LOD, and no additional exclusion based on a separate LOD threshold was performed in the downstream analysis. The mean intra-plate coefficient of variation (%CV) across panels was 5.09% ± 3.39%. Because all samples were analyzed on a single plate, inter-plate CV was not applicable. For the same reason, no additional batch-effect correction was performed. NPX values were generated and normalized using Olink’s standard workflow. As platform-level reference information, a previous technical evaluation of Olink Explore 3072 reported high analytical precision, with a median technical CV of 6.3% and a mean technical CV of 9.8% [4]

**Sample Size and Power Analysis**

Based on previous meta-analyses showing effect sizes (Cohen's d) of 0.25-0.32 for cognitive improvements following multicomponent exercise programs, we calculated a required sample size of 46 participants (power = 0.90, α = 0.05) using G*Power 3.1 software.

**Effects Size**

Cohen’s d effect sizes were computed for all pairwise comparisons to quantify the magnitude of differences independent of sample size. Following a mixed-effects modeling approach, we defined $d=\frac{\Delta}{\hat{\sigma}}$, where Δ denotes the EMM difference from the model and $\hat{\sigma}$ is the residual standard deviation from the fitted mixed-effects model. To illustrate the magnitude of the within-group effect, absolute effect sizes (Cohen’s d) were also reported as absolute values when a significant change was observed in one group (e.g., intervention) but not in the other (e.g., control).

Reference

1 Hakoda Y, Sasaki M. Group version of the stroop and reverse-stroop test. *Jpn J Educ Psychol*. 1990;38:389–94. doi: 10.5926/jjep1953.38.4_389

2 Cansino S, Estrada-Manilla C, Hernández-Ramos E, *et al.* The rate of source memory decline across the adult life span. *Dev Psychol*. 2013;49:973–85. doi: 10.1037/a0028894

3 Zafarana A, Lenatti C, Hunt L, *et al.* Visual perceptual learning is enhanced by training in the illusory far space. *Q J Exp Psychol (Hove)*. 2025;78:1077–87. doi: 10.1177/17470218241256870

4 Sissala N, Babačić H, Leo IR, *et al.* Comparative evaluation of Olink Explore 3072 and mass spectrometry with peptide fractionation for plasma proteomics. *Commun Chem*. 2025;8:327. doi: 10.1038/s42004-025-01753-2
