# Supplementary material for: Preliminary effects of a four-month circuit training intervention on cognitive function and exploratory plasma proteomic profiles in middle-aged and older women: an open-label randomized controlled trial
Source: Front Sports Act Living. 2026 Jun 23;8:1851134. doi: 10.3389/fspor.2026.1851134 (PMC13337939; doi:10.3389/fspor.2026.1851134)
Supplement: Supplementary file 2 [file Supplementaryfile2.docx]

**Secondary Outcomes**

**Gut Microbiome**

Analysis

Alpha diversity indices (Shannon index, Chao1 estimator, and Simpson index) were analyzed using Kruskal-Wallis tests with pairwise comparisons. Beta diversity (Bray-Curtis dissimilarity) was analyzed using the Analysis of Similarities (ANOSIM) to test for differences in microbial community composition between groups and time points. The relative abundance of bacterial taxa at the phylum and genus levels as well as short-chain fatty acid (SCFA) concentrations (acetate, propionate, and n-butyrate) were log-transformed when necessary to achieve normality and analyzed using paired t-tests within each group to compare the pre- and post-intervention values.

Microbial Diversity

Analysis of the gut microbiota diversity showed no significant intervention effects on either alpha or beta diversity. Regarding alpha diversity, no significant differences were observed between the baseline and post-intervention time points for the Shannon index, Chao1 estimator, or Simpson index in either the exercise or control groups (all p > 0.05, Kruskal-Wallis test). Similarly, beta diversity analysis using Bray-Curtis dissimilarity revealed no significant differences in the overall microbial community composition between the groups or time points.

Short-Chain Fatty Acids (SCFAs)

Analysis of SCFA concentrations (acetate, propionate, and n-butyrate) revealed no significant main effects of time or group × time interactions on any of the measured SCFAs (all p > 0.05).

The implications of these changes in the gut microbiota extend beyond simple bacterial composition shifts to potential systemic health benefits. Exercise interventions promote beneficial alterations in gut microbiota composition, including enhanced microbial diversity and enrichment of health-associated bacterial taxa, effects that align with the established anti-inflammatory properties of regular physical activity [1]. This implies that circuit training may modulate the gut-brain axis by reducing microbial-induced inflammation. Previous findings have also indicated that habitual physical activity increases SFCAs levels [2]. Short-chain fatty acids (SCFAs) are thought to enhance cognitive function via the brain–gut axis, partly through vagal-nerve signaling and neurotransmitter modulation [3].

Physical function was assessed using handgrip strength measurement, a 5-meter walk test, and single-leg support tests. Psychological characteristics were evaluated using validated Japanese versions of questionnaires, including the Short Grit Scale [4], Brief Resilience Scale [5]. Mental health was assessed using the Kessler Psychological Distress Scale (K6) [6]. Quality of life was measured using the SF-36 Health Survey [7]. Physical activity levels were assessed using the Global Physical Activity Questionnaire (GPAQ) [8], and mood states were evaluated using the Profile of Mood States (POMS) [9]. Additional measures included sleep quality (Pittsburgh Sleep Quality Index) [10,11], constipation assessment [12], and basic demographic and health information.

Analysis of secondary outcomes revealed several improvements over the 16-week intervention period, although no significant group × time interactions were observed for any measure (all p > 0.05). Post-hoc within-group comparisons following the significant main effects of time showed the following changes:

Physical Activity: Both groups demonstrated significant increases in leisure-time high-intensity physical activity (exercise group, p = 0.0354; control group, p = 0.0241).

Quality of Life: The exercise group showed significant improvement in role limitations caused by emotional problems, as measured by the SF-36 (p = 0.0257).

Sleep Quality: Subjective sleep quality improved significantly in both groups (exercise group, p = 0.0071; control group, p = 0.0225).

No significant changes were observed in other secondary measures, including physical function, psychological characteristics, and mood states (all p > 0.05).

References

1 Hawley JA, Forster SC, Giles EM. Exercise, the gut microbiome and gastrointestinal diseases: Therapeutic impact and molecular mechanisms. *Gastroenterology*. 2025;169:48–62. doi: 10.1053/j.gastro.2025.01.224

2 Aya V, Flórez A, Perez L, *et al.* Association between physical activity and changes in intestinal microbiota composition: A systematic review. *PLoS One*. 2021;16:e0247039. doi: 10.1371/journal.pone.0247039

3 Dalile B, Van Oudenhove L, Vervliet B, *et al.* The role of short-chain fatty acids in microbiota-gut-brain communication. *Nat Rev Gastroenterol Hepatol*. 2019;16:461–78. doi: 10.1038/s41575-019-0157-3

4 Nishikawa K, Okugami S, Amemiya T. Development of the Japanese short grit scale (grit-S). *Pasonariti Kenkyu*. 2015;24:167–9. doi: 10.2132/personality.24.167

5 Tokuyoshi Y, Morita M. Development and validation of the Brief Resilience Scale. *Psychol Assoc Jpn*. 2015;1EV-055.

6 Furukawa TA, Kawakami N, Saitoh M, *et al.* The performance of the Japanese version of the K6 and K10 in the World Mental Health Survey Japan. *Int J Methods Psychiatr Res*. 2008;17:152–8. doi: 10.1002/mpr.257

7 Fukuhara S, Bito S, Green J, *et al.* Translation, adaptation, and validation of the SF-36 Health Survey for use in Japan. *J Clin Epidemiol*. 1998;51:1037–44. doi: 10.1016/s0895-4356(98)00095-x

8 Bull FC, Maslin TS, Armstrong T. Global physical activity questionnaire (GPAQ): nine country reliability and validity study. *J Phys Act Health*. 2009;6:790–804. doi: 10.1123/jpah.6.6.790

9 Heuchert JP, McNair DM. Profile of mood states 2nd edition^TM^. PsycTESTS Dataset. 2012.

10 Doi Y, Minowa M, Uchiyama M, *et al.* Psychometric assessment of subjective sleep quality using the Japanese version of the Pittsburgh Sleep Quality Index (PSQI-J) in psychiatric disordered and control subjects. *Psychiatry Res*. 2000;97:165–72. doi: 10.1016/s0165-1781(00)00232-8

11 Buysse DJ, Reynolds CF 3rd, Monk TH, *et al.* The Pittsburgh Sleep Quality Index: a new instrument for psychiatric practice and research. *Psychiatry Res*. 1989;28:193–213. doi: 10.1016/0165-1781(89)90047-4

12 Nomura H, Agatsuma T, Mimura T. Validity and reliability of the Japanese version of the Patient Assessment of Constipation Quality of Life questionnaire. *J Gastroenterol*. 2014;49:667–73. doi: 10.1007/s00535-013-0825-y
