# Supplementary material for: Preliminary effects of a four-month circuit training intervention on cognitive function and exploratory plasma proteomic profiles in middle-aged and older women: an open-label randomized controlled trial
Source: Front Sports Act Living. 2026 Jun 23;8:1851134. doi: 10.3389/fspor.2026.1851134 (PMC13337939; doi:10.3389/fspor.2026.1851134)
Supplement: Supplementary file 3 [file Supplementaryfile3.docx]

**Proteomic analysis**

Proteomic analysis was conducted using multiple complementary approaches. First, paired t-tests were performed within each group to identify the proteins showing significant changes from baseline to post-intervention. Volcano plots were generated using paired t-tests with significance thresholds set at p < 0.05 and |log2 fold change| > 0.5 to visualize differentially expressed proteins. Between-group comparisons of protein changes (post-baseline differences) were performed using unpaired t-tests. For functional annotation, significantly altered proteins (identified by volcano plot analysis) were subjected to pathway enrichment analyses. Gene Ontology (GO) enrichment analysis was performed using the clusterProfiler package to identify overrepresented biological processes, molecular functions, and cellular components. Kyoto Encyclopedia of Genes and Genomes (KEGG) pathway analysis was performed to examine the metabolic and signaling pathways, whereas Reactome pathway analysis provided insights into biological reactions and pathways. UniProt IDs were converted to Entrez Gene IDs using the org.Hs.eg.db database for pathway analyses. Enrichment significance was set at p < 0.05, with Benjamini-Hochberg correction for multiple comparisons (q < 0.2).

Supplemental Figure 1 illustrates the proteomic results for the exercise group, including (A) a volcano plot of protein expression changes and (B–D) the results of Gene Ontology (GO), Kyoto Encyclopedia of Genes and Genomes (KEGG), and Reactome pathway enrichment analyses, respectively. Supplemental Figure 2 shows the corresponding analyses for the control group. Proteomic analysis was conducted on plasma samples from 44 participants who completed the 16-week intervention (22 in the exercise group and 22 in the control group). Of the original 23 exercise group participants who completed the study, 1 was excluded from the proteomic analysis for having the lowest attendance rate among all participants, despite meeting the minimum target of 80% attendance. From the original 25 control group participants who completed the study, 3 were excluded: 1 because of hemolyzed blood samples during collection and 2 because of an inability to complete the cognitive assessment (Flanker task). As this study aimed to examine protein-cognition relationships, participants who could not perform the Flanker task, the least demanding cognitive task in our battery, were excluded to ensure valid cognitive-proteomic associations. Pre- and post-intervention plasma samples from 44 participants were analyzed using a comprehensive proteomic platform.

**Differentially Expressed Proteins**

Volcano plot analysis was performed to identify differentially expressed proteins within each group from the baseline to post-intervention using paired t-tests. Of the 1,098 proteins analyzed, significant changes were observed for 273 proteins in the exercise group (p < 0.05) and 436 proteins in the control group (p < 0.05).

Supplemental Figures 1(a) and 2 (a) illustrate the exercise and control conditions, respectively.

**Gene Ontology (GO) Analysis**

GO enrichment analysis was performed on 111 altered proteins in the exercise group and 275 proteins in the control group. In the exercise group, GO analysis identified 426 significantly enriched biological processes (q < 0.05, Benjamini-Hochberg correction). The most highly enriched pathways included positive regulation of the MAPK cascade, vesicle fusion, and organelle membrane fusion. Other notably enriched processes involved endosomal transport, membrane fusion, and positive regulation of NF-κB signaling.

The control group showed 577 enriched biological processes, with enriched pathways related to phosphatidylinositol 3-kinase/protein kinase B signal transduction, cellular response to chemical stress, and response to oxidative stress. Supplemental Figures 1(b) and 2 (b) illustrate the exercise and control conditions, respectively.

**KEGG Pathway Analysis**

KEGG pathway analysis identified 189 and 257 enriched pathways in the exercise and control groups, respectively (q < 0.05). In the exercise group, the most highly enriched pathway was SNARE interactions in vesicular transport, followed by Yersinia infection and NF-kappa B signaling pathway. Other notably enriched pathways included the MAPK signaling pathway, TNF signaling pathway, and regulation of actin cytoskeleton.

The control group showed similar top-enriched pathways, with SNARE interactions in vesicular transport, Yersinia infection, and NF-kappa B signaling pathway among the most significant. Further, the control group showed enrichment of the lipid and atherosclerosis pathway and the PD-L1 expression and PD-1 checkpoint pathway in cancer. Supplemental Figures 1(c) and 2 (c) illustrate the exercise and control conditions, respectively.

**REACTOME Pathway Analysis**

REACTOME pathway analysis identified 656 enriched pathways in the exercise group and 950 enriched pathways in the control group (q < 0.05). In the exercise group, the most highly enriched pathways included intra-Golgi traffic, CDC42 GTPase cycle, and TRAF6-mediated NF-κB activation. Other notable pathways involved ERBB2 signaling, the RHO GTPase cycle, and parasite infection.

The control group showed different top-enriched pathways, with ERBB2 signaling and downregulation of ERBB2 signaling as the most significant. Additionally, platelet activation, signaling and aggregation, PI3K/AKT signaling in cancer, and NF-κB activation pathways were prominently enriched. Supplemental Figures 1(d) and 2(d) illustrate the exercise and control conditions, respectively.

Proteomic analysis revealed differences in protein expression patterns between the exercise and control groups at the pre- and post-training time points, providing molecular-level insights into the systemic effects of circuit training. Notably, the alteration in the exercise group suggests that exercise may promote protein expression stability and homeostatic maintenance. This finding is in contrast with the extensive protein changes observed in the control group, which may reflect age-related physiological drift or stress-induced dysregulation. Pathway enrichment analyses further elucidated these differences, with the exercise group showing enrichment in fundamental cellular processes, including SNARE-mediated vesicular transport, membrane fusion, and GTPase signaling pathways. These pathways are essential for synaptic function, intracellular trafficking, and cellular communication [1,2], suggesting that exercise promotes optimal cellular machinery function. In contrast, the control group exhibited prominent enrichment in inflammation- and stress-related pathways, including NF-κB signaling, oxidative stress responses, and disease-associated pathways, such as lipid, atherosclerosis, and cancer-related signaling [3].


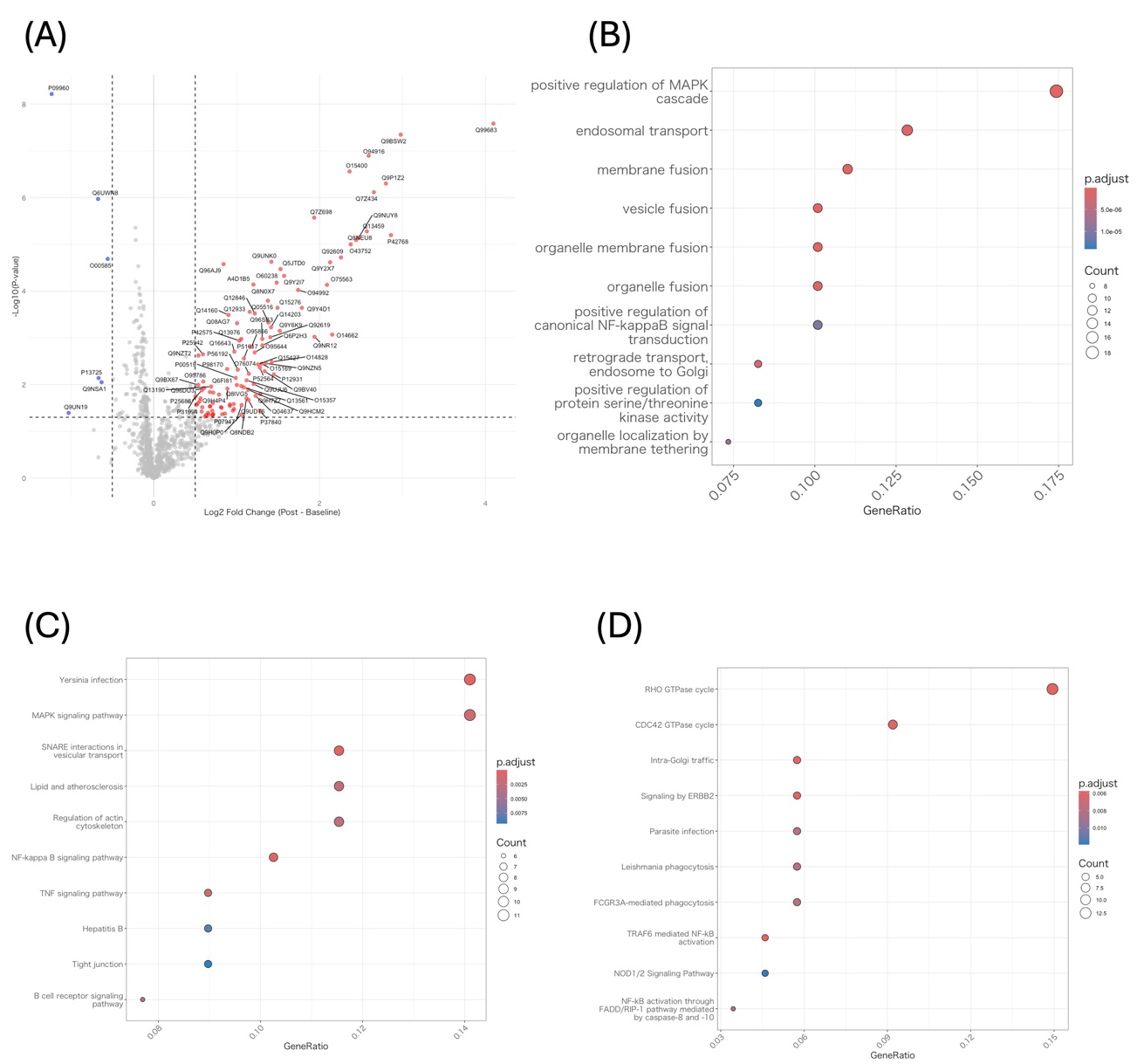


Supplemental Figure 1: Proteomic analysis results in the exercise intervention group. (A) Volcano plot illustrating protein expression changes between post- and pre-intervention. (B) Gene Ontology (GO) enrichment analysis of differentially expressed proteins. (C) Kyoto Encyclopedia of Genes and Genomes (KEGG) pathway analysis. (D) Reactome pathway analysis.


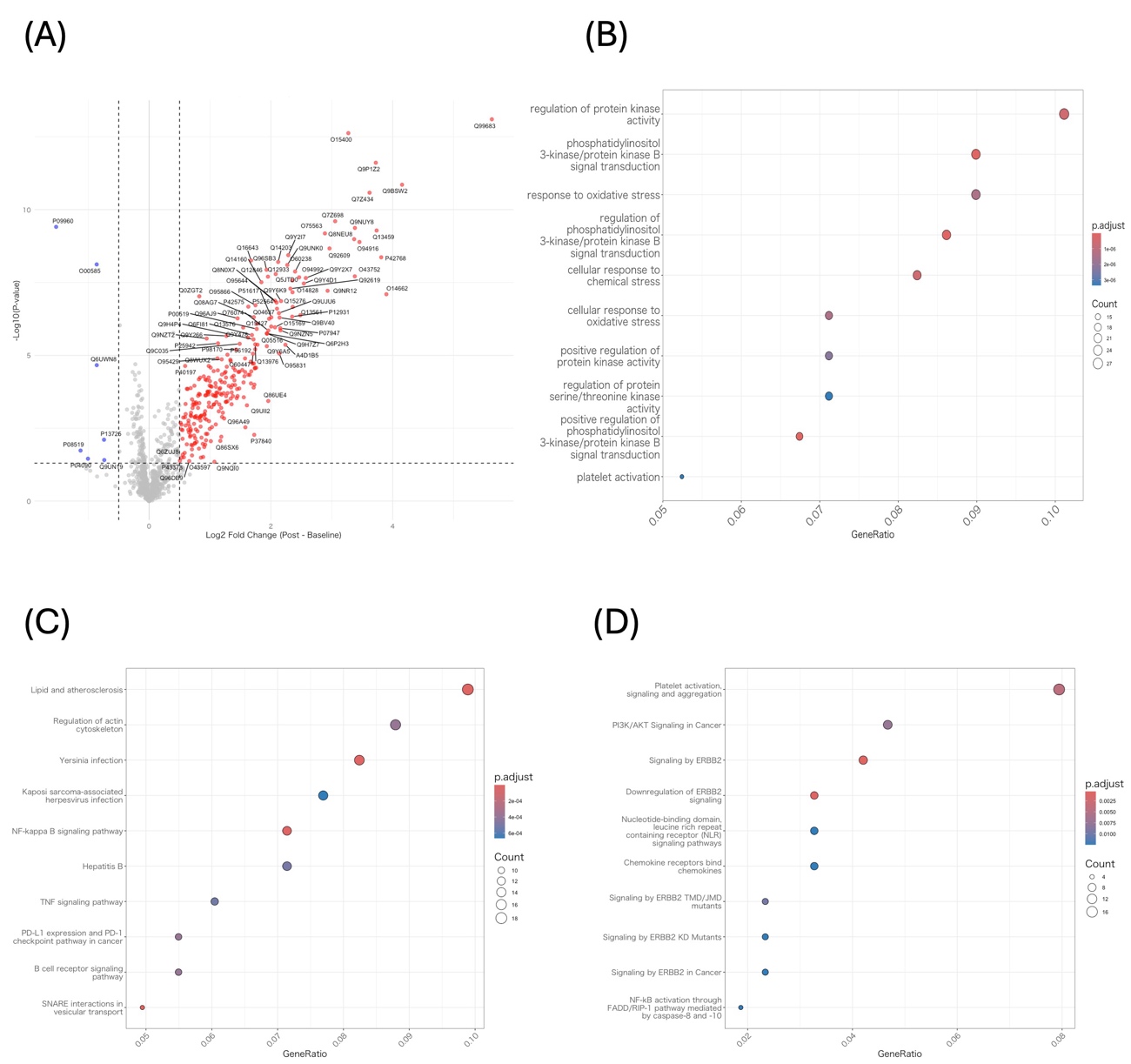


Supplemental Figure 2: Proteomic analysis results in the control group. (A) Volcano plot illustrating protein expression changes between post- and pre-intervention. (B) Gene Ontology (GO) enrichment analysis of differentially expressed proteins. (C) Kyoto Encyclopedia of Genes and Genomes (KEGG) pathway analysis. (D) Reactome pathway analysis.


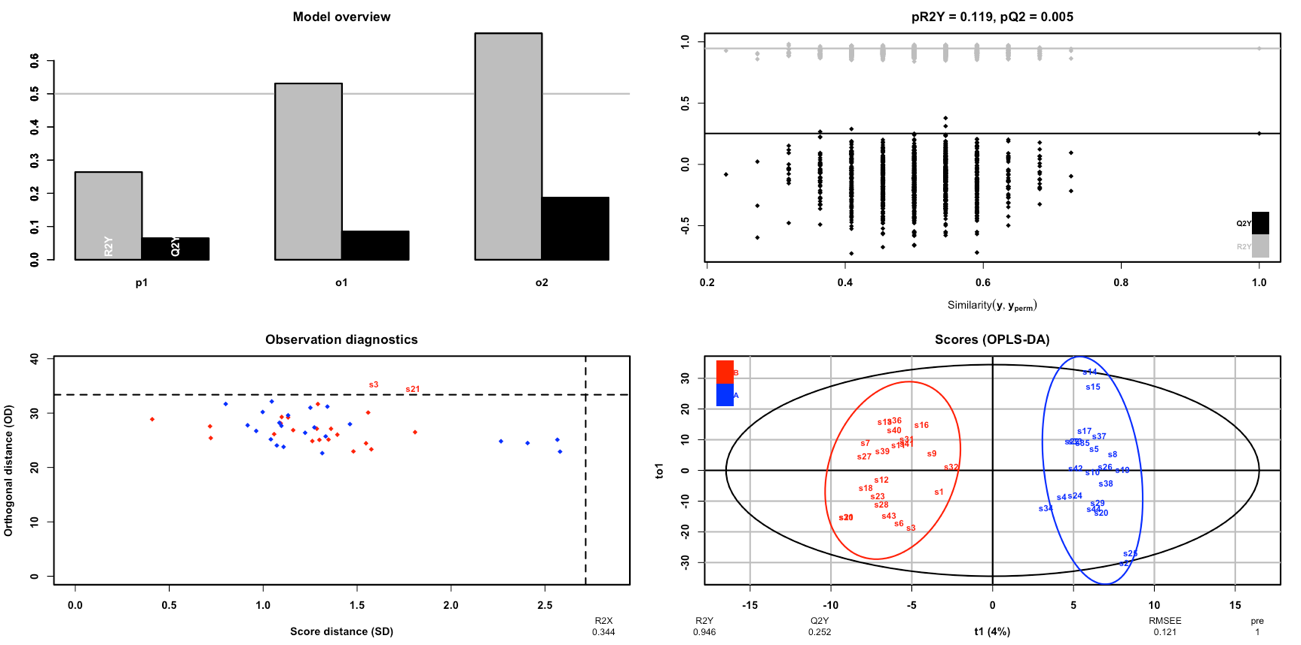


Supplemental Figure 3: Permutation Test (n=1000) Validating the OPLS-DA Model

References

1 Lambert NA, Johnston CA, Cappell SD, *et al.* Regulators of G-protein signaling accelerate GPCR signaling kinetics and govern sensitivity solely by accelerating GTPase activity. *Proc Natl Acad Sci U S A*. 2010;107:7066–71. doi: 10.1073/pnas.0912934107

2 Jahn R, Cafiso DC, Tamm LK. Mechanisms of SNARE proteins in membrane fusion. *Nat Rev Mol Cell Biol*. 2024;25:101–18. doi: 10.1038/s41580-023-00668-x

3 Guo Q, Jin Y, Chen X, *et al.* NF-κB in biology and targeted therapy: new insights and translational implications. *Signal Transduct Target Ther*. 2024;9:53. doi: 10.1038/s41392-024-01757-9
